# Co-regulatory expression quantitative trait loci mapping: method and application to endometrial cancer

**DOI:** 10.1186/1755-8794-4-6

**Published:** 2011-01-12

**Authors:** Kenneth S Kompass, John S Witte

**Affiliations:** 1 Department of Epidemiology and Biostatistics and Institute for Human Genetics, University of California, San Francisco, San Francisco, California, USA

## Abstract

**Background:**

Expression quantitative trait loci (eQTL) studies have helped identify the genetic determinants of gene expression. Understanding the potential interacting mechanisms underlying such findings, however, is challenging.

**Methods:**

We describe a method to identify the *trans-*acting drivers of multiple gene co-expression, which reflects the action of regulatory molecules. This method-termed *co-regulatory expression quantitative trait locus *(creQTL) *mapping*-allows for evaluation of a more focused set of phenotypes within a clear biological context than conventional eQTL mapping.

**Results:**

Applying this method to a study of endometrial cancer revealed regulatory mechanisms supported by the literature: a creQTL between a locus upstream of STARD13/DLC2 and a group of seven IFNβ-induced genes. This suggests that the Rho-GTPase encoded by STARD13 regulates IFNβ-induced genes and the DNA damage response.

**Conclusions:**

Because of the importance of IFNβ in cancer, our results suggest that creQTL may provide a finer picture of gene regulation and may reveal additional molecular targets for intervention. An open source R implementation of the method is available at http://sites.google.com/site/kenkompass/.

## Background

Expression Quantitative Trait Locus (eQTL) mapping searches across the genome for markers associated with individual transcripts to identify loci containing regulatory elements [1,2]. Although *cis *regulators are easily interpreted, assigning function to *trans *regulators is more difficult. At the transcriptional level, genes in *trans *are often co-regulated by transcription factors binding the same regulatory elements in the noncoding sequences of multiple genes [3]. When looking genome-wide, however, genes across many ontologies acting upstream of transcription factors participate in the co-regulation of genes [4,5]. Because of the difficulty in predicting the *trans-*regulators, the majority of transcriptional regulatory proteins remain unknown.

Identification of genetic components of gene co-regulation is important because there is compelling evidence that aberrant gene co-regulation participates in human disease [6,7]. Investigations into the genetic basis of gene co-regulation have used Bayesian networks to identify *cis*- and *trans-*acting factors controlling modules of co-regulated genes [8-10] or gene clusters. A key result here was the identification of associations that would have been missed when genes were tested individually, as in traditional eQTL. Biologically, this is very compelling because genes typically do not perform their functions in isolation but rather in coordinated groups. The existing Bayesian methods have focused primarily on the identification of yeast regulatory programs where other sources of information, such as sequence conservation, transcription factor binding site (TFBS) data, or protein interaction data are readily available and serve as prior information [8,10]. Extension of these methods to human genetics with data from HapMap subjects has shown that sequence conservation and *cis-*regulatory information were the most useful prior data [8].

Studies in other human cohorts and mice have used directed Bayesian networks or undirected weighted gene coexpression networks to incorporate existing marker and phenotype data into models that have made biologically validated predictions [11-16]. These network-based methods and their alternatives (e.g. [17-21]) show great promise but often have high computational complexity, making them most practical for smaller datasets with limited numbers of traits. Furthermore, prior information is generally not readily available in humans. For example, most TF binding sites remain unknown and even within the same tissue, the vast majority of TFBS appear divergent between human and mouse [22]. This suggests that relying on sequence conservation or the presence of conserved TF binding motifs may miss some key associations and that agnostic, complementary methods should be developed.

Many algorithms that search for the determinants of gene co-regulation assign each gene to a single cluster (e.g. [4,9,23]), which is limiting, because genes can belong to different clusters under different biological conditions [24]. More recent network approaches overcome this problem by examining differential canonical correlation between multiple states, such as healthy and diseased, or with a reference [11]; these approaches, however, may rely on methods that are not robust to non-normal data to find correlated genes. This may be a problem for gene microarray expression data, which is often not normally distributed. Alternatively, robust, "mega-clustering" methods have been developed to provide improved estimates of co-regulation for microarray data [25]. One such algorithm-the 'Gene Recommender'-has successfully predicted previously unknown interactions that were verified experimentally in a multicellular organism [26]. A key property of the Gene Recommender is the categorization of genes into clusters under different conditions (i.e., allowing for "biclusters") where different samples' contributions to the given cluster may vary. Since inexpensive genotyping platforms can presently interrogate >1 million SNPs and we are rapidly shifting into the era of whole genome sequencing, existing genetics and systems biology methods would be nicely complemented by computationally feasible, agnostic approaches to the detection of *trans*-acting factors that regulate groups of genes.

Therefore, here we extend the Gene Recommender with an approach that can systematically identify *trans *loci controlling gene co-regulation. We broadly refer to the identification of gene co-expression trait loci as 'co-regulatory expression quantitative trait loci' (creQTL) mapping. Unlike *trans-*eQTL analysis, our method does not consider individual transcripts but rather focuses on multiple co-regulated transcripts. We provide a genome-wide implementation of the Gene Recommender and a statistical framework for association testing. The key steps of the creQTL approach are: gene clustering; calculation of each sample's similarity to each cluster; and statistical testing of how well genotype explains the similarity. We applied creQTL to a study of germline variants and tumor expression in endometrial cancer [27] and identified many loci significantly associated with gene co-regulation. These loci were commonly in noncoding regions closely in *cis *with genes encoding proteins required for transcription, signaling, cell adhesion, and development. Our results suggest that associating genetic variants with co-regulation via creQTL mapping provides an efficient and agnostic avenue for detecting biological factors important in the coordinate regulation of groups of genes.

## Methods

### creQTL Approach

To identify *trans *regulatory associations, creQTL mapping tests for the association between genetic variants spanning the genome and clusters of co-regulated genes. The variant data can be obtained, for example, from single nucleotide polymorphism (SNP) arrays. Gene expression data can be obtained from standard gene expression arrays or other high-throughput methods for mRNA quantification.

Gene clustering is based on a recursive, heuristic implementation of the Gene Recommender (GR) [26], automated for genome-wide application. GR is a rank-based biclustering algorithm with an existing, open source R implementation [28]. Here we outline relevant portions of the GR. First, gene expression data are normalized to a uniform distribution with mean zero and variance 1/3. A suggested gene list, based on prior knowledge, of at least two putative co-regulated genes is input to GR. For each putative cluster input, each sample's relative score, the "Z_E_(j)", is computed, as in Equation 1.

(1) $$Z_{E(j)}=\sqrt{k_{j}}\frac{{\bar{Y}}_{Q,j}}{\sqrt{{\hat{V}}_{Q,j}+\frac{1}{3p^{2}}}}$$

where *k_j _*is the number of gene expression values in sample *j, p *is the number of samples, and ${\bar{Y}}_{Q,j}$ is the median gene cluster expression value in sample *j*. Note that the original implementation of Gene Recommender used the mean gene cluster expression value in the numerator of Z_E_(j) [26]; later versions of the algorithm used the median [28]. Here we used the latest version. ${\hat{V}}_{Q,j}$ is the sample variance, given by

(2) $${\hat{V}}_{Q,j}=\frac{k_{j}-1}{k_{j}}\mathrm{var}(Y_{Q,j})$$

where var(*Y_Q, j_*) is the variance of the genes within the gene cluster in sample *j *(both equations, ref. [26]). Z_E_(j) has an approximate Student's t null distribution and larger values indicate tighter co-regulation of clustered genes within sample *j*. Once Z_E_(j) has been computed, an incremental approach is used to compute the correlation coefficient, s.g.i., with a scoring function that minimizes the number of nonquery genes scoring higher than those in the query. The final s.g.i., which is proportional to the Euclidean distance, is then based on the most informative experiments.

It is not computationally feasible to compute all possible gene clusters from the dataset with GR, so heuristics are necessary to find tightly co-regulated gene clusters. Our extensions permit genome-wide use of the GR by automatically selecting putative co-regulated genes, running the algorithm, and then recursively modifying the query using leave-one-out cross validation (LOOCV) as a scoring function until the cluster converges at a point where all query genes contribute approximately equally to the cluster. The procedure is as follows. First, using each gene as a seed for a potential cluster, initial predictions of co-regulated genes are made using Spearman's rank correlation. If the number of highly correlated genes is less than 5 or more than 20, the putative cluster is expanded or trimmed to the most correlated 5 or 20 genes, respectively. Next, the Gene Recommender algorithm is run with this initial gene cluster, and then rerun using the top hits (by s.g.i, the Gene Recommender normalized correlation metric) from the initial run. If the seed gene scores highly after the second run (i.e., within the top 50 genes most correlated with the putative cluster) LOOCV is used to trim the cluster to only the highest scoring hits (regardless of gene set size). Once a tightly regulated cluster has been found, the next-highest scoring genes are added incrementally while stringently keeping LOOCV scores low. Ultimately, all genes within a predicted cluster will have an approximately equivalent contribution to the cluster, as determined by LOOCV.

Once gene clusters were assigned, for creQTL association testing, we computed a modified version of Z_E_(j), called Z2_E_(j), to describe how tightly regulated the gene cluster was within a given sample. The modified version replaces the numerator of Z_E_(j) with the mean of all genes' contributions to the cluster and adds a small positive constant, *s0*, to the denominator in order to moderate variance and avoid extremely large values of Z2_E_(j). *s0 *was chosen by minimizing the coefficient of variation of the denominator of Z_E_(j) across moving windows of data, akin to the strategy of Tusher et al. [29] for the moderation of t statistics. The modified statistic for association testing is

(3) $$Z2_{E(j)}=\sqrt{k_{j}}\frac{mean({\bar{Y}}_{Q,j}Y_{Q,j})}{\sqrt{{\hat{V}}_{Q,j}+\frac{1}{3p^{2}}}+s0}$$

In place of the median expression value in the numerator of the original Z_E_(j), we multiplied the sample's median gene expression value with the expression values for each individual gene within the cluster, and computed the mean of the resulting vector. Although this strategy will produce a higher Z2_E_(j) in the case of a cluster with strongly driven outliers, it will also produce a lower Z2_E_(j) in the presence of weakly driven outliers, even when the cluster is otherwise strongly driven. Thus, a larger priority was given to penalizing strongly driven clusters with weakly driven outliers than the opposite. Overall, this produced smaller values of Z2_E_(j) and gave a slightly greater dispersion of clusters with outliers when compared to taking the median gene expression value (not shown). Our software implementation provides a choice of either calculation for the numerator.

The association between genotypes and the Z2_E_(j) statistic was then evaluated with Bartlett's k-sample variance test [30], using the 'bartlett.test' function in R to find significant differences in the variance of Z2_E_(j) across each genotype using additive coding (i.e. AA, AB, BB). Resulting p-values were then adjusted for FDR using the 'p.adjust' function in R and the method of Benjamini and Hochberg [31].

### Application of creQTL to Endometrial Cancer

We applied creQTL mapping to a study of 52 endometrial tumor samples with genotype and expression data available (NCBI GEO 14860; [27]). We downloaded the raw genotype and normalized microarray data from GSE14860. Genotype data were from Affymetrix 100K SNP chips, and calls were made with the 'crlmm' function from the 'oligo' library (v1.10.0) [32] for Bioconductor (v2.5; [33]) and R (v2.10.0; [34]). Prior to association testing, SNP markers were prefiltered to satisfy the following stringent conditions for each marker: a call confidence value of at least 95% in 51/52 samples, no significant deviations from Hardy-Weinberg equilibrium at p = 0.05 (with 'snpMatrix' v1.8.0 [32]), and at least two different genotypes observed with at least 5 samples for each genotype. This was analogous to requiring a SNP minor allele frequency of 5% if two genotypes were observed, and 14% if all three genotypes were seen. To enrich the microarray data for abundant and strongly-hybridized transcripts, we calculated the variance of each probe and excluded the lowest 50% from our analyses. Microarray data were ranked and normalized to mean 0 and variance 1/3 across each gene, the default for the Gene Recommender algorithm. SNP r^2 ^values were calculated with 'snpMatrix.' Because all 52 samples were from a single county in Norway [27], we did not perform any adjustments for population stratification.

For comparison with the creQTL results, we also undertook an eQTL analysis of the EC dataset. Here, the microarray data were normalized to have a mean of zero and variance equal to one within each individual sample. *cis *eQTL analysis evaluated all SNPs within 5 MB of a gene using an analysis of variance (ANOVA) of expression level on genotype and including covariates identified with surrogate variable analysis [35]. *trans *eQTL was done similarly, focusing on the remaining SNPs > 5 MB from any protein-coding gene. For *cis *and *trans *eQTL analyses, p-values were adjusted separately for FDR [31].

Finally, we evaluated whether associated genes were overrepresented in broad gene ontology categories for creQTL. We organized all Gene Ontology [36] annotations into Biological Process (BP), Cellular Component (CC), and Molecular Function (MF) subcategories (Affymetrix, Santa Clara CA). SNPs were assigned to genes, as for *cis-*eQTL, using the minimum q-value for association across all tested SNPs within a 5 MB distance cutoff (to generate per-gene q-values). For all GO subcategories with at least 10 genes, the two-sample Kolmogorov-Smirnov test (one-tailed) was used to test for enrichment of significant genes in each Gene Ontology category. Specifically, to compute exact p-values for each category, we compared the observed q-value for a given gene set to those calculated from 10,000 same-sized sets of randomly drawn q-values. Because of the large number of categories, many of which contained overlapping genes, resulting p-values (for each GO category) were then adjusted for FDR [31].

## Results

**Figure 1:**
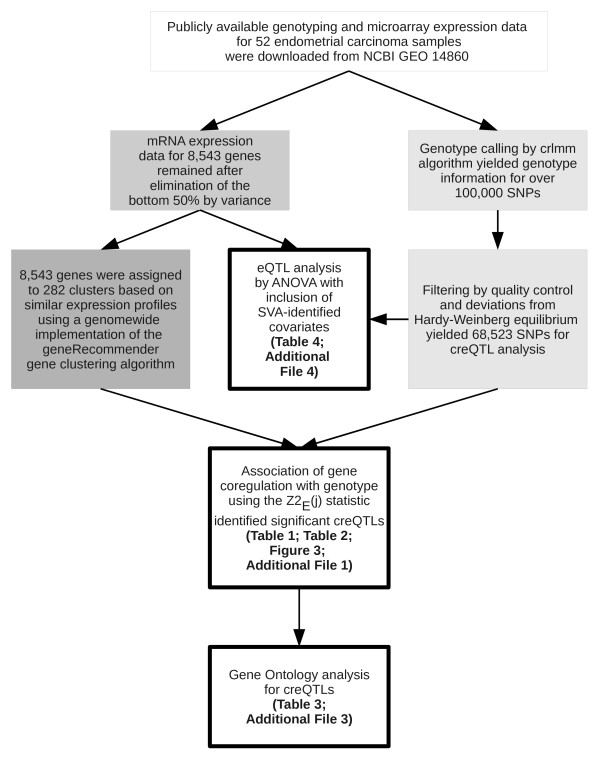
**Overview of analysis**.

The key steps in our creQTL approach and application to identify loci controlling co-regulation of genes are outlined in Figure 1. Because of the overall unreliability of low-expression measurements in gene microarray expression data and our interest only in genes with dynamic expression patterns, we prefiltered those data to exclude the bottom 50% of probes by variance, leaving 8,543 for inclusion in our analysis of gene clustering and QTL testing. 68,523 SNPs from the Affymetrix 100K chip met cutoffs for inclusion in our analysis.

**Figure 2:**
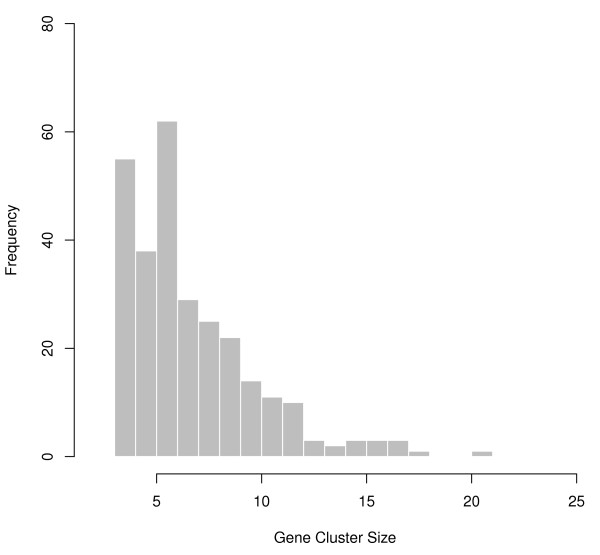
**Histogram of gene cluster sizes identified from the EC gene expression data**.

From the expression data, we identified 282 gene clusters with at least 3 co-regulated genes. The distribution of gene cluster sizes is shown in Figure 2. Many genes present within the same clusters had overlapping, known biological functions, and genes within individual clusters often were syntenic, such as the IFNβ-induced genes *IFI44, IFI44L*, and *IFIT1*, *-2*, and *-3*, on chromosomal regions 1p31.1 and 10q23-26, contained within a cluster significantly associated with a SNP/locus upstream of *RFC3 *and *STARD13*.

**Figure 3:**
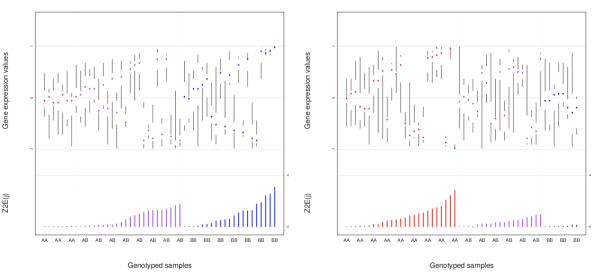
**creQTL1 (left panel) and creQTL2 (right panel) from Table 1, with SNP IDs rs9315220 and rs2296697, respectively**. For each association, co-regulation of a given cluster and a SNP is shown. The 52 endometrial carcinoma samples are color coded by genotype on the x-axis (upper panel), with every third sample labeled beneath the axis. On the y-axis (upper panel), normalized gene cluster expression values are plotted. The median, 25th/75th quantiles, minimum, and maximum gene values across all genes in the cluster are shown for each sample. The bottom panel of each figure shows the Z2_E_(j) statistic for each sample and the given cluster.

We found 453 associations between genotype and Z2_E_(j) with q-values < = 0.005 (Additional File 1 contains annotated associations) after 19,323,486 variance tests of 282 gene clusters and 68,523 SNPs. Note that a FDR q-value of 0.005 corresponded to an unadjusted p-value of approximately 1 × 10^-7^. Table 1 and Figure 3 highlight two of the most significant creQTLs that were within 5 MB of a protein-coding gene. As shown in Figure 3, for statistically significant creQTLs, the SNP genotypes exhibit varying contributions to the clusters. Moreover, samples that contribute most to a given cluster have more extreme, more tightly-clustered expression values; samples that contribute little to a given cluster have expression values (and contributions) closer to zero with greater variability. Additional File 2 shows the q-value density for associations as a function of gene cluster size. As can be seen in the figure, there was no relationship between q-values and gene cluster size.

**Table 1:** Two representative, significant creQTL associations.

creQTL 1					
SNP Annotation					
Locus	dbSNP	Location (bp)	Position	Gene Symbol	Gene Title
chr13q12.3-q13	rs9315220	304602	upstream	*RFC3*	replication factor C (activator 1) 3, 38 kDa
chr13q12-q13	rs9315220	227702	upstream	*STARD13*	StAR-related lipid transfer (START) domain containing 13
					
**Gene Cluster Annotation**					
**Locus**	**Entrez ID**	**q_creQTL_**	**p_eQTL_**	**Gene Symbol**	**Gene Title**
chr1p31.1	10561	1.13 × 10^-4^	0.21	*IFI44*	interferon-induced protein 44
chr1p31.1	10964		0.28	*IFI44L*	interferon-induced protein 44-like
chr10q23-q25	3433		0.21	*IFIT2*	interferon-induced protein with tetratricopeptide repeats 2
chr10q24	3437		0.49	*IFIT3*	interferon-induced protein with tetratricopeptide repeats 3
chr10q25-q26	3434		0.19	*IFIT1*	interferon-induced protein with tetratricopeptide repeats 1
chr12q24.1	4938		0.52	*OAS1*	2',5'-oligoadenylate synthetase 1, 40/46 kDa
chr21q22.3	4599		0.56	*MX1*	myxovirus (influenza virus) resistance 1, interferon-inducible protein p78 (mouse)
					
**creQTL 2**					
**SNP Annotation**					
**Locus**	**dbSNP**	**Location (bp)**	**Position**	**Gene Symbol**	**Gene Title**
chr1p31.1	rs2296697	0	intron	*LPHN2*	latrophilin 2
					
**Gene Cluster Annotation**					
**Locus**	**Entrez ID**	**q_creQTL_**	**p_eQTL_**	**Gene Symbol**	**Gene Title**
chr2p25	6664	5.40 × 10^-4^	0.92	*SOX11*	SRY (sex determining region Y)-box 11
chr3p14.3	57408		0.38	*LRTM1*	leucine-rich repeats and transmembrane domains 1
chr11q22.3	4317		0.10	*MMP8*	matrix metallopeptidase 8 (neutrophil collagenase)
chr14q11.2	9362		0.38	*CPNE6*	copine VI (neuronal)
chr21q22.3	54058		0.64	*C21orf58*	chromosome 21 open reading frame 58
NA	NA		0.35	NA	NA

For each of the two creQTLs shown in this table (labeled '**creQTL 1**' and '**creQTL 2**'), the associated SNP (labeled '**SNP Annotation**') is first shown with annotation for the nearest coding genes in *cis*. Beneath the SNP annotation is annotation for the significantly associated, co-regulated gene cluster (labeled '**Gene Cluster Annotation**'). These two significant associations correspond to the two loci shown in Figure 3. '**Position**' indicates the relative location of the SNP relative to the nearest coding gene; '**Location (bp)**' indicates the physical distance, in base pairs, between the SNP and the nearest coding genes; **p_eQTL _**indicates unadjusted significance of standard eQTL analysis.

For the association between rs9315220 and the cluster noted above, several other nearby SNPs (rs9315219, rs9315215, rs7981602, rs4943110) were also significantly associated with this cluster (see Additional File 1), reflecting the high linkage disequilibrium (LD) among them (the pairwise r^2 ^across all five SNPs ranged from 0.92 to 0.96 in these 52 samples). The smallest q-value was nearest to the *STARD13 *locus (q-value = 1.13 × 10^-4^). Another strong association (q = 5.40 × 10^-4^) shown in Figure 2 was between the intronic SNP rs2296697 in *LPHN2 *(latrophilin 2) and a cluster of genes including *C21orf58*, *CPNE6*, *SOX11*, *MMP8*, *LRTM1*, and one unannotated gene. The genes within this cluster have established roles in Ca^2+^-dependent signaling and cancer [37-41] (**Discussion**). Interestingly, a conventional *trans*-eQTL analysis between the SNPs and individual expression values of these co-regulated genes did not detect any associations (see Table 1); these results were typical of other clusters (data not shown).

**Figure 4:**
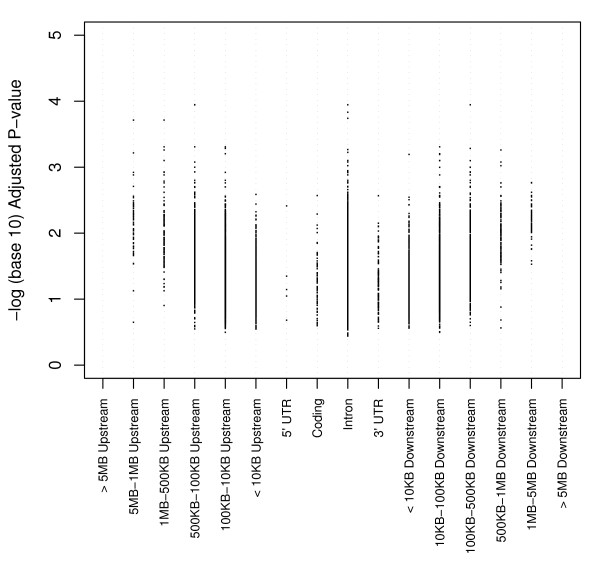
**Genewise genomic locations of SNP q-values**. After assigning 68,523 SNPs to 8,398 genes based on the minimum association q-value within 5 MB of the nearest gene's coding sequence, q-values were binned into categories based on their relative position to the nearest gene. Note that the q-values do not reach the bottom of the plot as they were chosen as the *minimum *value within 5 MB of the nearest gene.

The strongest regulatory loci for creQTL (q = 5.50 × 10^-4^) are shown in Table 2. We calculated pairwise r^2 ^between all 453 SNPs from creQTLs with q < 0.005 and eliminated those with r^2 ^values above 0.5, leaving 338 creQTLs. Of the 338 SNPs remaining, 190 were in noncoding regions; 145 were intronic; two were in coding sequences; and one was in the 3' untranslated region. Interestingly, many of the associated creQTLs contained SNPs in noncoding sequences >500 kb from the nearest gene. After assigning the 68,523 SNPs to 8,398 protein-coding genes in *cis *(**Methods**), we plotted the resulting 8,398 q-values versus *cis-*location relative to the nearest gene in Figure 4. Consistent with the pattern for the highest-scoring hits at q < 0.005, associations appeared most enriched in the intronic regions, and within the non-intronic, noncoding regions, significant associations appeared most in regions >10 KB away from the coding sequence.

**Table 2:** Top scoring creQTLs at a significance threshold of q = 5.50 × 10^-4^.

Locus	dbSNP	Location (bp)	Position	Gene Symbol	Gene Title	q_creQTL_
chr6q16	rs959186	125994	downstream	*RNGTT*	RNA guanylyltransferase and 5'-phosphatase	1.13 × 10-4
chr13q12.3-q13	rs9315215	348455	upstream	*RFC3*	replication factor C (activator 1) 3, 38 kDa	1.13 × 10-4
chr13q12-q13	rs9315215	183849	upstream	*STARD13*	StAR-related lipid transfer (START) domain containing 13	1.13 × 10-4
chrXq21.33	rs4969656	695921	upstream	*DIAPH2*	diaphanous homolog 2 (Drosophila)	1.93 × 10-4
chrXq21.3-q22	rs4969656	2315179	upstream	*NAP1L3*	nucleosome assembly protein 1-like 3	1.93 × 10-4
chr3q13.1	rs4856121	2246645	upstream	*ALCAM*	activated leukocyte cell adhesion molecule	1.93 × 10-4
chr5q31-q32	rs319217	0	intron	*PPP2R2B*	protein phosphatase 2 (formerly 2A), regulatory subunit B, beta isoform	1.13 × 10-4
chr1p31.1	rs2296697	0	intron	*LPHN2*	latrophilin 2	5.40 × 10-4
chr2p21	rs222471	31099	downstream	*KCNG3*	potassium voltage-gated channel, subfamily G, member 3	4.91 × 10-4
chr2p21	rs222471	49701	upstream	*COX7A2L*	cytochrome c oxidase subunit VIIa polypeptide 2 like	4.91 × 10-4
chr4q21	rs1992489	59023	upstream	*CXCL13*	chemokine (C-X-C motif) ligand 13	5.19 × 10-4
chr4q21.1	rs1992489	282671	downstream	*CCNG2*	cyclin G2	5.19 × 10-4
chr14q13-q21	rs1951319	680177	downstream	*RPL10L*	ribosomal protein L10-like	5.48 × 10-4
chr14q21.2	rs1951319	717439	upstream	*C14orf106*	chromosome 14 open reading frame 106	5.48 × 10-4
chr8p23.2	rs1714757	0	intron	*CSMD1*	CUB and Sushi multiple domains 1	1.47 × 10-4
chr16p13.12	rs1159167	147599	upstream	*ERCC4*	excision repair cross-complementing rodent repair deficiency, complementation group 4	4.91 × 10-4
chr16p13.12	rs1159167	968670	upstream	*CPPED1*	calcineurin-like phosphoesterase domain containing 1	4.91 × 10-4
chr22q12.1	rs10483151	0	intron	*TTC28*	tetratricopeptide repeat domain 28	1.81 × 10-4

Associated gene clusters have been omitted for brevity and are listed in Additional File 1. '**Position**' and '**Location (bp)**' as for Table 1; **q_creQTL_**' indicates FDR-adjusted significance.

We further analyzed the creQTL results with Gene Ontology in order to determine if any broad categories of genes participated in the regulation of gene clusters. There were 648, 176, and 310 Gene Ontology Biological Process (BP), Cellular Component (CC), and Molecular Function (MF) annotations, respectively, for gene set enrichment testing that were represented on the SNP arrays after assigning the 68,523 SNPs to 8,398 protein-coding genes in *cis*, using an arbitrary minimum cutoff of 10 genes for a category to be tested. Top hits are shown in Table 3 at a more relaxed q = 0.05. The most significant ontologies were related to cell adhesion, potassium- and calcium ion transport, binding, channel activity, development, and the nervous system, particularly development and plasticity. For Biological Process and Molecular Function, as expected, some of the most enriched categories were related to transcription factors and other aspects of transcriptional regulation. For Molecular Function, categories '0003700' (transcription factor activity) and '0003714' (transcription corepressor activity), with 497 and 74 genes, respectively, were just beyond the FDR significance cutoff with adjusted p-values of 0.056 and 0.078 (Additional File 3). For Cellular Component, nearly all significant categories were directly related to various aspects or cell-type specific (e.g. neuronal) specializations of the cellular membrane (9 of 13 hits at q = 0.05).

**Table 3:** Gene Ontology enrichment analysis for creQTL at q = 0.05.

Gene Ontology Biological Process			
Category ID	Category Size	Category Title	q_KS_
7214	18	gamma-aminobutyric acid signaling pathway	< 0.008
7612	21	learning	< 0.008
7411	62	axon guidance	< 0.008
7156	69	homophilic cell adhesion	< 0.008
7417	80	central nervous system development	< 0.008
7155	320	cell adhesion	< 0.008
45944	203	positive regulation of transcription from RNA polymerase II promoter	0.008
7399	225	nervous system development	0.008
45595	12	regulation of cell differentiation	0.014
48754	19	branching morphogenesis of a tube	0.018
30900	42	forebrain development	0.018
45165	29	cell fate commitment	0.027
43065	69	positive regulation of apoptosis	0.04
7224	16	smoothened signaling pathway	0.043
7420	74	brain development	0.043
30326	35	embryonic limb morphogenesis	0.043
7169	56	transmembrane receptor protein tyrosine kinase signaling pathway	0.043
6813	94	potassium ion transport	0.043
			
**Gene Ontology Cellular Component**			
**Category ID**	**Category Size**	**Category Title**	**q_KS_**
5913	18	cell-cell adherens junction	< 0.004
45202	156	synapse	< 0.004
30054	231	cell junction	< 0.004
14069	49	postsynaptic density	0.004
45211	79	postsynaptic membrane	0.004
30424	83	axon	0.006
781	13	chromosome, telomeric region	0.013
5912	27	adherens junction	0.013
34707	36	chloride channel complex	0.016
5923	40	tight junction	0.016
5886	1504	plasma membrane	0.037
42734	21	presynaptic membrane	0.038
5578	174	proteinaceous extracellular matrix	0.038
			
**Gene Ontology Molecular Function**			
**Category ID**	**Category Size**	**Category Title**	**q_KS_**
8066	11	glutamate receptor activity	< 0.008
5216	187	ion channel activity	< 0.008
5509	491	calcium ion binding	< 0.008
4993	11	serotonin receptor activity	< 0.008
4970	12	ionotropic glutamate receptor activity	0.009
5234	12	extracellular-glutamate-gated ion channel activity	0.009
43565	273	sequence-specific DNA binding	0.009
5244	85	voltage-gated ion channel activity	0.016
4890	17	GABA-A receptor activity	0.017
5246	11	calcium channel regulator activity	0.019
31404	49	chloride ion binding	0.023
30594	18	neurotransmitter receptor activity	0.028
30165	32	PDZ domain binding	0.029
5001	13	transmembrane receptor protein tyrosine phosphatase activity	0.029
5267	53	potassium channel activity	0.029
5230	24	extracellular ligand-gated ion channel activity	0.037
5254	48	chloride channel activity	0.041
8146	26	sulfotransferase activity	0.041
16455	11	RNA polymerase II transcription mediator activity	0.042

Results are presented for Biological Process, Cellular Component, and Molecular Function, respectively. Category IDs are presented minus leading zeroes. '**q_KS_**' indicates FDR-adjusted significance of the category.

eQTL analysis identified no significant *cis-*eQTLs (q = 0.05); the smallest FDR-adjusted significance level for *cis-*eQTLs was q = 0.098 (not shown). There were 63 significant *trans-*eQTLs (q = 0.05; Additional File 4), and the most significant are shown in Table 4. A raw p-value of approximately 6 × 10^-9 ^corresponded to q = 0.05. Even with a high 50% FDR cutoff, there were only 1,293 *cis-*eQTLs (of 1,468,327), or 0.09%, and 290,625 (of 507,010,655), or 0.06% *trans-*eQTLs. Although marginally significant (q = 0.049), the association between *ZNF639 *and *TCF12 *is included in Table 4 due to association of 3q26 with survival in this dataset [27] (**Discussion**). We did not perform gene ontology analysis of the eQTL experiment since there were no clear *cis*-eQTLs detected.

**Table 4:** The most significant associations from *trans-*eQTL analysis at q = 0.05.

SNP Locus	dbSNP	Location (bp)	Position	SNP Gene Symbol	mRNA Locus	Entrez ID	mRNA Gene Symbol	q_eQTL_
chrXp22.11	rs4828879	23770	upstream	*PRDX4*	chr11p15.5	7140	*TNNT3*	0.003
chr7q32.3	rs277491	0	intron	*PLXNA4*	chr3p26-p25	7862	*BRPF1*	0.003
chr4q32.3	rs10517754	0	intron	*FSTL5*	chr22q12.1	25770	*C22orf31*	0.005
chr7q34	rs2363830	0	intron	*DENND2A*	chr11q23-q24	56	*ACRV1*	0.016
chr3q22.1	rs938243	0	intron	*CPNE4*	chr16q23.3	93517	*SDR42E1*	0.018
chr2q13	rs10496425	0	intron	*CCDC138*	chr7q11.23	7461	*CLIP2*	0.019
chr2q13	rs7591305	0	intron	*CCDC138*	chr7q11.23	7461	*CLIP2*	0.019
chr10q21.1	rs10509024	0	intron	*PCDH15*	chrXp11.23	778	*CACNA1F*	0.029
chr3p24.3	rs964910	561192	upstream	*SGOL1*	chr1q44	114548	*NLRP3*	0.029
chr21q21.1	rs2826728	0	intron	*NCAM2*	chr11p15.5	7140	*TNNT3*	0.029
chr21q21.1	rs2155798	0	intron	*NCAM2*	chr11p15.5	7140	*TNNT3*	0.029
chr1q43	rs2790645	135107	upstream	*CHRM3*	chr5q31-q32	5521	*PPP2R2B*	0.029
chr11q14.1	rs62388	0	intron	*DLG2*	chr7q34	154790	*CLEC2L*	0.029
chr3q26.33	rs9290675	34917	upstream	*ZNF639*	chr15q21	6938	*TCF12*	0.049

'**Position**' indicates SNP genomic location; ' **q_eQTL_**' indicates FDR-adjusted significance. Annotation is shown for SNPs in the first 5 columns, then for mRNA expression; e.g. for the first (non-header) row of this table, the mRNA expression levels for gene *TNNT3 *were significantly associated with genotype at rs4828879, which is 23,770 base pairs upstream of the *PRDX4 *locus.

## Discussion

creQTL provides an agnostic framework for predicting *trans *regulators of clusters of genes. It does not require any one particular biclustering method or statistical test and can be extended to any species for which genetic markers (including those derived from linkage) and gene expression data can be obtained; for example, creQTL mapping in mice would allow the study of gene regulation in many disease models and in normal biological processes such as development, which are highly relevant to human disease. Like eQTLs, creQTLs may be incorporated into additional analyses. As recent studies have used eQTL data to assemble genetic loci into directed, Bayesian networks to predict causal relationships (e.g. [16,42]), future studies could assemble creQTLs into directed or undirected gene networks. Because creQTL mapping groups co-regulated genes into a smaller number of modules, network construction using creQTL information in place of eQTL information should be computationally more efficient.

In our application to endometrial cancer, there was an abundance of creQTLs in noncoding regions and introns, and relatively very few in coding regions. Polymorphisms in coding regions, although observed less frequently, would likely generate more pronounced effects, and for signaling molecules, these could result in substantial downstream effects important for carcinogenesis due to a change in function of the protein. However, these alleles, which are likely to be under strong negative selection and very rare, would not be covered by the genotyping platform used in the present study. Unlike the results of eQTL studies, our strongest hits were not typically located in *cis *regions very close to the coding sequences of individual genes, but rather in more distant, noncoding regions located over 10 KB from the nearest gene. This suggests that regions that regulate clusters of genes, unlike those regulating individual genes, are located farther from coding sequences and may possibly function as general enhancers. However, without careful experimentation, such as promoter reporter gene assays that would specifically test the enhancer activity of these sequences, it is difficult to predict their actual roles. Future studies could address the relationship between location, allele frequency, and other empirical aspects of creQTLs in larger cohorts and healthy tissue. Collapsing SNPs into a dominant model would permit testing of less common SNPs, as requiring five observations in each genotype essentially restricted our analysis to more common variants.

Our results for creQTL from the Gene Ontology enrichment analysis indicated a regulatory hierarchy, consistent with data from wet-lab studies, where signal transduction molecules receive signals at the plasma membrane and transduce them through various intracellular intermediates to the nucleus to activate specific transcription factors, other DNA-binding proteins, and other transcriptional regulatory proteins. The relative abundances of DNA-binding proteins at the promoter region may control gene expression [43]. Because of the enrichment of cell adhesion and signaling ontologies at the cell junction and membrane at the highest significance levels, the major steps of gene regulation in endometrial cancer may occur not within the nucleus but rather at the cellular membrane. In addition, GO enrichment in genes required for telomere maintenance suggests additional target genes and loci for further investigation, as telomerase has long been considered an attractive target for therapy in endometrial carcinoma [44] and more recently, a mouse model of endometrial cancer showed that telomere length affected initiation of type II carcinogenesis [45].

Gene Ontology analysis is not without its own pitfalls. Besides the obvious effects of overlapping annotations for the majority of genes and annotation bias, the categories themselves often do not describe real biological phenomena. Given that transcription factors are very cell-type specific, a category that includes all of them is not particularly useful. In light of this, the q-values for transcription factor-related categories are likely to be overly conservative, and would probably be smaller if those TFs not expressed in this tissue were excluded. Future studies could address this problem using array and wet lab expression data.

For the association shown in Figure 2, the nearest coding sequence to rs9315220 is *STARD13*, a.k.a. *DLC2 *(deleted in liver cancer 2). All genes of this cluster but one (OAS1), including *MX1, IFI44, IFIT2, IFIT3, IFI44L*, and *IFIT1*, are known to be induced by IFNβ in humans[46]. IFNβ, besides its role in the treatment of multiple sclerosis (MS), is anti-tumorigenic [47-50]. Similarly, many genes from this cluster also have established or putative roles in cancer; *IFI44 *was part of a small set of genes independently validated for recurrence status in non-small cell lung carcinoma [37], is anti-proliferative in melanoma cell lines [38], and is upregulated in squamous cell carcinoma [39]. *IFIT2 *inhibits migration and proliferation in squamous cell carcinoma cultures [41]. *IFIT1 *was one of a small group of genes that predict carcinogenesis after training on DNA-damage response [40], while *STARD13 *is upregulated in human lymphocytes following gamma-irradiation [51], suggesting that *IFIT1 *may be a downstream target of *STARD13 *following DNA damage.

*STARD13/DLC2 *encodes a Rho GTPase activating protein and was identified from a region of chromosome 13 exhibiting a loss of heterozygosity in hepatocellular carcinoma [52]. It is a tumor suppressor that antagonizes Rho expression [53], and its downregulation or deletion has been reported in multiple cancers [54,55]. Supporting its role as a regulator of IFNβ-induced genes, an intronic SNP in *STARD13 *was amongst eighteen that were replicated in a study of responders to IFNβ therapy in MS patients [56]. Further, SNPs in *OAS1 *have been associated with MS susceptibility [57]. Therefore, this particular gene cluster and its predicted regulatory partner, *STARD13*, may be interacting determinants of the response to various negative stimuli. This also proposes the hypothesis that the role of *STARD13 *as a tumor suppressor is from its induction in response to DNA damage, and that deletion of this region in at least ten different cancers is pathogenic possibly due to hyper-activation of Rho, which promotes migration, proliferation, and invasion [58]. In this case, beyond improvement of the total lack of significant association from the individual eQTLs, creQTL proposed a more complete picture of the biological pathway.

We detected a strong association of rs2296697, within an intron of *LPHN2 *(latrophilin 2), with a cluster of genes including *C21orf58, CPNE6, SOX11, MMP8*, and *LRTM1*. *LPHN2 *belongs to the latrophilin subfamily of G-protein coupled receptors, with known roles in cell adhesion and signal transduction [59]. Cell adhesion was among the most enriched categories from Gene Ontology analysis of creQTL. *CPNE6 *(copine VI), is a member of the ubiquitous copine family, which bind phospholipid in Ca^2+^-dependent manner and have roles in cellular division and growth [60]. Although very little is known regarding *CPNE6*, other members of the copine family have been shown to promote tumor cell migration [61] and repress *NF-KB *transcription [62]. *SOX11 *is a TF with important roles in brain development that is strongly upregulated in lymphoma [63] and malignant glioma, where it may affect tumorigenesis [64,65]. *SOX11 *may also be a prognostic marker for recurrence-free survival in another uterine neoplasia, epithelial ovarian cancer [66]. *MMP8 *(human neutrophil collagenase) is a member of the matrix metallopeptidase family. Recent work has shown that a SNP in *MMP8 *may be a predictor of lung cancer risk [67], and that higher plasma levels of *MMP8 *may protect against lymph node metastasis in breast cancer [68]. Consistent with these findings, somatic mutations of *MMP8 *were common in melanomas, and mutated *MMP8 *failed to inhibit tumor formation *in vivo *[69], suggesting widespread roles for *MMP8 *in cancer progression. Three members of this gene cluster have no known roles, yet the functions of the other cluster members suggest they could contribute to cancer progression. In this case, the association of this cluster of genes with *LPHN2 *has suggested that *LPHN2 *may be a regulatory point for the modulation of genes with important roles in cancer, including *MMP8 *and *SOX11*.

eQTL analysis was not done in the original paper presenting this dataset [27], and the general lack of findings from the eQTL analysis may be from a lack of power, given that there were only 52 samples. This is not surprising: an eQTL study of 60 samples from the CEU cohort in the HapMap data identified only 10 *cis-*eQTLs and 94 in *trans *[70], although there may have been some technical issues here [71].

However, in the present study, creQTL appeared to identify interactions that were supported by recent work in the literature. A very compelling benefit of creQTL over *trans-*eQTL is greater computational efficiency and a reduced multiple testing burden, as there was an approximate 25-fold reduction in the number of statistical tests. By organizing genes into co-regulated clusters prior to statistical testing, the problem becomes more computationally feasible and the resulting output more biologically interpretable. The identification of more significant hits after adjustment was likely not simply due to a lighter multiple testing penalty; *cis-*eQTL required 1,468,327 tests, far less than the 19,323,486 tests for creQTL, and produced no statistically significant results even at the more liberal q-value cutoff of 0.05. Overall, these results suggest that individual SNP-gene interactions are more difficult to detect (as in eQTL) when compared to relative changes in the expression levels of tightly co-regulated genes, in agreement with previous work in yeast [9]. creQTL may be better-suited to the identification of the strongest *trans *drivers of gene expression because it better explains the data; i.e., genes do not work individually to exert their biological effects, but rather in tightly coordinated groups. However, our results regarding the location of these drivers of co-regulation relative to coding sequences suggests that they might be disjoint from *cis *drivers of individual gene expression.

Some *trans*-eQTL hits were significant and may merit further investigation based on previously established biological roles for associated loci, indicating complementary utility of eQTL and that the two methods may be most useful when applied side-by-side. Copy number analysis of this EC dataset by the original authors revealed two regions of gain that were predictive of survival - 3q26.32 and 12p12.1 [27]. Deletions were not considered. Because *PIK3CA *is located in this region, the authors proposed a role for the PI3-kinase pathway, and found supporting evidence for this theory based on indirect, bioinformatic analysis of existing, *in vitro *data. Located 200 kb from *PIK3CA *is *ZNF639*, a.k.a. *ZASC1*, which is often contained within the same amplicon in cancer [72,73]. *ZNF639 *was originally identified in squamous cell carcinoma as a Kruppel-like transcription factor with mRNA expression levels prognostic for survival and metastasis [72,73]. *trans-*eQTL identified a SNP less than 35 kb upstream of *ZNF639 *(SNP_A-1686963) driving expression of the *TCF12 *gene at 15q21. *TCF12*, a.k.a. *HTF4 *or *ME1*, is a basic helix-loop-helix TF with key roles in development [74]. In a mouse experiment, *TCF12 *was associated with obesity [14], a key prognostic factor for endometrial cancer [75]. Because the stoichiometric concentration of TFs within the nucleus may control gene expression [43], this association proposes a link between obesity and *TCF12 *through *ZNF639*. Because *ZNF639 *regulates anchoring of E-cadherin to the cytoskeleton through alpha N-catenin [76], given the importance of cell adhesion proteins in cancer [77], future studies of endometrial cancer might focus on *ZNF639*.

## Conclusions

With the advent of low-cost SNP and expression arrays, human genetics has become a common tool for the dissection of gene regulation. Genetic approaches to the study of transcriptional regulation are compelling because they can detect regulatory molecules at all stages of gene regulation. Our approach expands upon previous methods and uses genetic variation to help identify transcriptional regulatory mechanisms, providing a biologically intuitive approach for detecting potential links between genotype and gene co-regulation.

## List of Abbreviations

ANOVA: analysis of variance; BP: gene ontology biological process; CC: gene ontology cellular component; creQTL: co-regulatory expression quantitative trait locus; EC: endometrial cancer; EDF: empirical distribution-free; eQTL: expression quantitative trait locus; FDR: false discovery rate; GO: gene ontology; KS: Kolmogorov-Smirnov test; LD: linkage disequilibrium; LOOCV: leave-one-out cross-validation; MF: gene ontology molecular function; mRNA: messenger ribonucleic acid; MS: multiple sclerosis; SNP: single nucleotide polymorphism; TF: transcription factor; TFBS: transcription factor binding site.

## Competing interests

The authors declare that they have no competing interests.

## Authors' contributions

Conceived and designed the experiments: KSK and JSW. Analyzed the data: KSK. Wrote software: KSK. Wrote the paper: KSK and JSW. Both authors read and approved the final manuscript.

## Pre-publication history

The pre-publication history for this paper can be accessed here:

http://www.biomedcentral.com/1755-8794/4/6/prepub

## Supplementary Material

Additional file 1 **Significant creQTLs**. All significant creQTLs at q = 0.005 with annotation. Click here for file

Additional file 2 **creQTL q-values by gene cluster size**. Plot of q-values for all associations, grouped by gene cluster size. The right panel is a zoomed view of the left panel, highlighting the rejection region. For both panels, the legend at upper left indicates the number of genes in the cluster. Click here for file

Additional file 3 **creQTL Gene Ontology**. All results of Gene Ontology testing for creQTL. Click here for file

Additional file 4 ***trans-*eQTL**. Results of *trans-*eQTL analysis at q = 0.05, annotated. Click here for file
