# Supplementary figures and images for: Co-regulatory expression quantitative trait loci mapping: method and application to endometrial cancer
Source: BMC Med Genomics. 2011 Jan 12;4:6. doi: 10.1186/1755-8794-4-6 (PMC3032645; doi:10.1186/1755-8794-4-6)

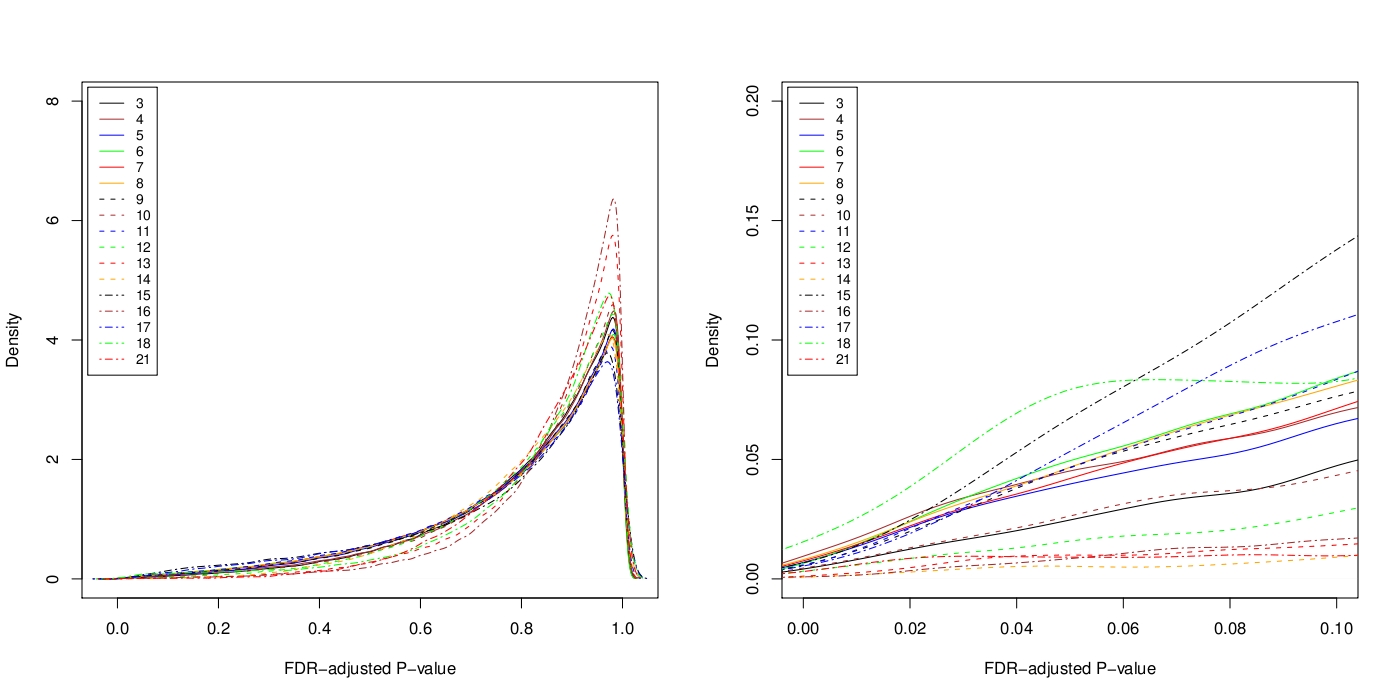

Supplement: Additional file 2 — creQTL q-values by gene cluster size. Plot of q-values for all associations, grouped by gene cluster size. The right panel is a zoomed view of the left panel, highlighting the rejection region. For both panels, the legend at upper left indicates the number of genes in the cluster. [file 1755-8794-4-6-S2.ZIP › Add.File.2.jpeg]

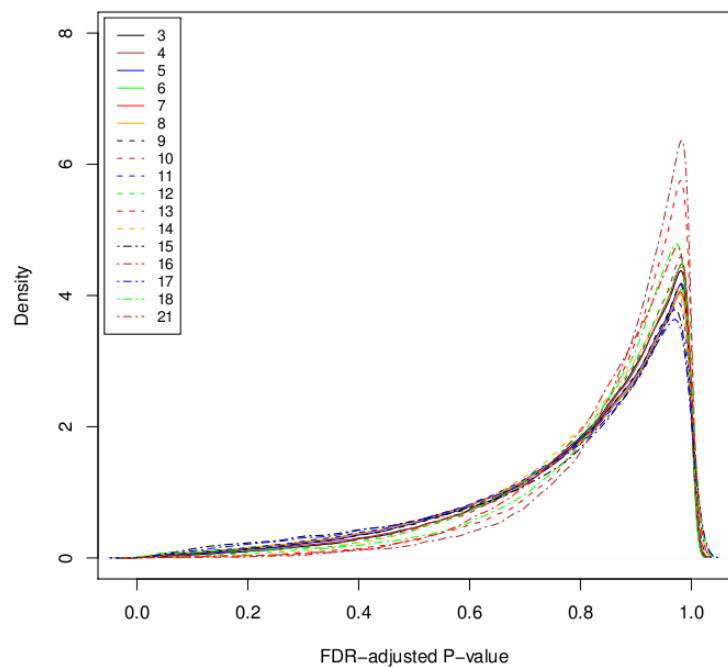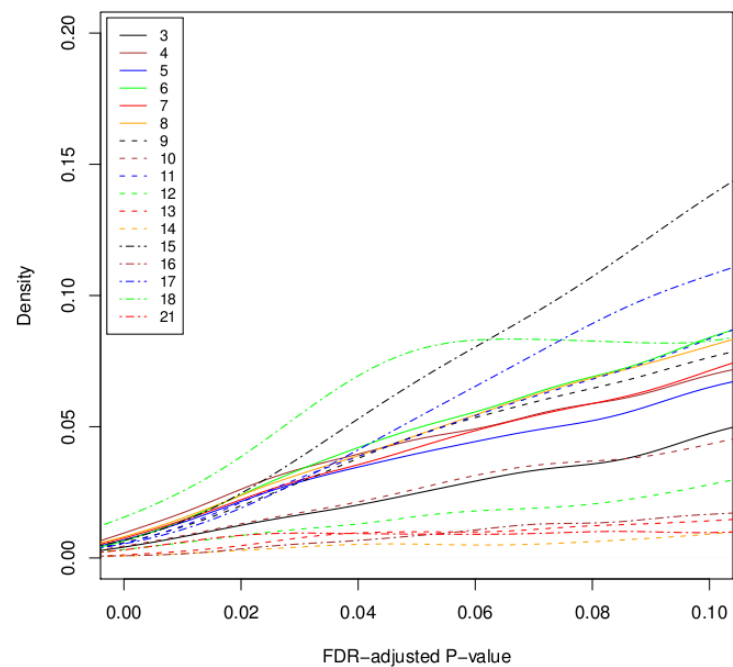

Supplement: Additional file 2 — creQTL q-values by gene cluster size. Plot of q-values for all associations, grouped by gene cluster size. The right panel is a zoomed view of the left panel, highlighting the rejection region. For both panels, the legend at upper left indicates the number of genes in the cluster. [file 1755-8794-4-6-S2.ZIP › Add.File.2.pdf]
